# GenProb‐PCSM: A Simplified Weighted Germline Score for Prostate Cancer‐Specific Mortality

**DOI:** 10.1002/pros.70244

**Published:** 2026-09-08

**Authors:** Jun Wei, Zhuqing Shi, Lucy Lu, John Wang, Julian Xu, Ashley J. Mulford, Huy Tran, Annabelle Ashworth, S. Lilly Zheng, Alan R. Sanders, William B. Issacs, Jun Luo, Brian Helfand, Jim Lu, Jianfeng Xu

**Affiliations:** ^1^ Program for Genomic Translational Research Endeavor Health Evanston Illinois USA; ^2^ GoPath Diagnostics Buffalo Grove Illinois USA; ^3^ Genomic Health Initiative Endeavor Health Evanston Illinois USA; ^4^ University of Chicago Pritzker School of Medicine Chicago Illinois USA; ^5^ James Buchanan Brady Urological Institute, Johns Hopkins School of Medicine Baltimore Maryland USA; ^6^ Division of Urology Endeavor Health Evanston Illinois USA

**Keywords:** DNA damage repair genes, germline genetics, KLK3, metastatic progression, Prostate cancer‐specific mortality (PCSM)

## Abstract

**Background:**

We previously developed a tier‐based germline classification using the National Comprehensive Cancer Network (NCCN)‐recommended DNA damage repair (DDR) genes and *KLK3* I179T to predict prostate cancer (PCa)‐specific mortality (PCSM). To provide an easier‐to‐use single inherited risk score while preserving gene‐specific effects, we developed GenProb‐PCSM.

**Methods:**

We analyzed 14,644 men with incident PCa from the UK Biobank. The cohort was randomly divided into training (60%) and independent testing (40%) datasets. GenProb‐PCSM was developed in the training cohort by integrating pathogenic variants in ten NCCN‐recommended DDR genes and the *KLK3* I179T variant using gene‐specific weights derived from Fine–Gray competing‐risk models. Performance was evaluated in the independent testing cohort by discrimination, calibration, and risk stratification. Secondary analyzes evaluated metastatic progression and the composite endpoint of metastatic progression and/or PCSM.

**Results:**

Among 14,644 men with incident PCa, 1,581 died from PCa. GenProb‐PCSM remained significantly associated with PCSM in the independent testing cohort (HR per SD, 1.18; 95% CI, 1.12–1.24; *p* < 0.001). Using predefined risk thresholds derived from the training cohort, patients in the intermediate‐ and high‐risk groups had significantly increased risks of PCSM compared with the low‐risk group (HR 1.49, 95% CI 1.22–1.84; and HR 4.04, 95% CI 2.58–6.33, respectively). GenProb‐PCSM also predicted independent metastatic progression and the composite endpoint of metastatic progression and/or PCSM.

**Conclusions:**

GenProb‐PCSM transforms complex germline findings into a single inherited risk score for PCSM and metastatic progression. Its simplicity and preservation of gene‐specific effects may facilitate clinical implementation of germline prognostic assessment in PCa.

## 1 Introduction

Prostate cancer (PCa) is the most commonly diagnosed cancer among men in many countries and a leading cause of cancer‐related death [1, 2]. Although most patients have indolent disease and excellent long‐term survival [3], a subset develops metastatic disease, which remains largely incurable despite advances in systemic therapies and is the primary cause of PCa‐specific mortality (PCSM) [4]. Identifying men with an inherited predisposition to lethal PCa has important clinical implications for treatment selection, surveillance intensity, and precision prevention and management [5, 6]. From a biological perspective, germline susceptibility is expected to influence the continuum from aggressive tumor biology to metastatic progression and ultimately PCSM [7]. Therefore, evaluating germline predictors across these clinically relevant outcomes may provide a more comprehensive assessment of inherited risk for lethal PCa [8, 9].

Accumulating evidence demonstrates that germline pathogenic variants (PVs) in DNA damage repair (DDR) genes contribute to lethal PCa, with *BRCA2* representing the strongest known genetic risk factor [7, 8, 9, 10, 11, 12, 13]. These DDR genes participate in homologous recombination repair, mismatch repair, and other pathways that preserve genomic integrity. Pathogenic variants may promote genomic instability, aggressive tumor biology, and disease progression. However, PVs in these individual genes are uncommon, limiting their utility for population‐level prognostic assessment. To address this challenge, we recently developed a clinically oriented germline risk stratification framework that classified National Comprehensive Cancer Network (NCCN)‐recommended DDR genes into Tier‐1 (*BRCA2*, *MSH6*, and *PALB2*) and Tier‐2 (*ATM, BRCA1, CHEK2, MLH1, MSH2, PMS2*, and *TP53*) groups according to their associations with PCSM and incorporated the common *KLK3* I179T variant as an independent prognostic marker [14]. The *KLK3* I179T variant complements the DDR genes through a distinct biological mechanism. Functional studies suggest that this missense variant alters PSA enzymatic activity, providing a biologically plausible explanation for its association with PCSM beyond its established relationship with PSA levels and prostate cancer detection [15].

However, interpretation of the tier‐based framework requires clinicians to integrate three separate genetic components (Tier‐1, Tier‐2, and *KLK3*), which may increase the complexity of routine clinical implementation. Furthermore, the tier‐based framework assigns equal weights to all genes within the same tier despite differences in the magnitude of risk across individual genes. To overcome these limitations, we developed GenProb‐PCSM (Genetic Probability of Prostate Cancer–Specific Mortality), a weighted germline risk score that integrates the gene‐specific effects of PVs in the 10 NCCN‐recommended DDR genes together with the common *KLK3* I179T variant into a single continuous measure of inherited risk. Rather than classifying patients into broad genetic categories, GenProb‐PCSM assigns gene‐specific weights according to their associations with PCSM. This continuum framework is consistent with recent ACMG recommendations advocating a transition from binary carrier status toward quantitative genetic risk assessment to better capture the spectrum of inherited disease risk and facilitate clinical decision‐making [16].

In this study, we developed and internally validated GenProb‐PCSM using PCSM as the primary endpoint in the UKB. We further evaluated whether GenProb‐PCSM predicts metastatic progression as a mutually exclusive clinical endpoint representing an earlier stage in the continuum of lethal disease, as well as the composite endpoint of metastatic progression and/or PCSM. We hypothesized that a weighted continuous germline score would provide an easier‐to‐use, more accurate, and clinically meaningful approach for inherited risk stratification than the tier‐based framework, thereby facilitating future clinical implementation of germline prognostic assessment in PCa.

## 2 Methods

Methods related to the UK Biobank (UKB) cohort, phenotype definitions, and germline variant annotation have been described previously [14]. Briefly, participants were men with incident PCa from the UKB, a population‐based cohort of individuals aged 40–69 years at recruitment [17]. PCa diagnoses (ICD‐10 code C61), metastatic disease (ICD‐10 codes C77–C79), and PCSM were ascertained through linkage to national cancer registries, hospital records, and death registry records. Men diagnosed with PCa before UKB recruitment were excluded.

Rare pathogenic or likely pathogenic (P/LP) variants in the 10 NCCN‐recommended DDR genes previously associated with PCSM (*BRCA2, MSH6, PALB2, ATM, BRCA1, CHEK2, MLH1, MSH2, PMS2*, and *TP53*) were evaluated [14]. Variants were classified according to ACMG/AMP criteria [18, 19, 20]. The common missense variant, *KLK3* I179T (rs17632542), was also evaluated [21].

Methods specific to the development and evaluation of GenProb‐PCSM are described below. The incident PCa cohort was randomly divided into a training cohort (60%) and an independent testing cohort (40%). GenProb‐PCSM was developed exclusively in the training cohort by integrating P/LP variants in the 10 NCCN‐recommended DDR genes together with the common *KLK3* I179T variant into a weighted continuous germline score. Gene‐specific weights were derived from the coefficients (log subdistribution hazard ratios) estimated from Fine–Gray competing‐risk models for PCSM, using age as the underlying time scale with delayed entry at age of PCa diagnosis. All models were adjusted for the first 10 genetic ancestry principal components (PCs). Individual GenProb‐PCSM scores were calculated by summing the weighted contributions of each genetic factor carried by a participant. Predicted cumulative PCSM probability by age 75 years was estimated for each participant, and empirically derived cutoffs defining low‐, intermediate‐, and high‐risk groups were determined exclusively in the training cohort. The resulting model coefficients and predefined risk thresholds were then applied without modification to the independent testing cohort for validation.

The primary endpoint was PCSM, which was used to develop GenProb‐PCSM. Secondary analyzes evaluated metastatic progression as a mutually exclusive endpoint in which patients experiencing PCSM were excluded, allowing assessment of whether GenProb‐PCSM predicts an earlier stage in the continuum of lethal disease. A third analysis evaluated the composite endpoint of metastatic progression and/or PCSM.

Model discrimination, calibration, and risk stratification were evaluated in the independent testing cohort. Calibration was assessed by comparing predicted and observed cumulative incidence of PCSM by age 75 years. Risk stratification was illustrated using cumulative incidence curves according to predefined GenProb‐PCSM risk groups. Discrimination was quantified using time‐dependent C‐indices, and differences between models were evaluated by bootstrap resampling (1000 replicates). Additional analyzes evaluated the performance of GenProb‐PCSM for predicting metastatic progression and the composite endpoint.

## 3 Results

### 3.1 Incident PCa Patients and Clinical Outcomes

Among 14,644 men diagnosed with incident PCa after UKB recruitment, 601 (4.10%) had metastatic disease at diagnosis and 14,043 (95.90%) had localized disease (Figure 1). For the primary analysis, 1581 men (10.8%) died from PCa, including 251 who had metastatic disease at diagnosis and 1330 who were initially diagnosed with localized disease. Secondary analyzes were restricted to patients who were initially diagnosed with localized PCa. In this cohort, 856 men subsequently developed metastatic progression in the absence of PCSM (i.e., independent of PCSM). Overall, 2186 men experienced the composite endpoint of metastatic progression and/or PCSM. Baseline demographic and clinical characteristics are summarized in Table 1.

**Figure 1:**
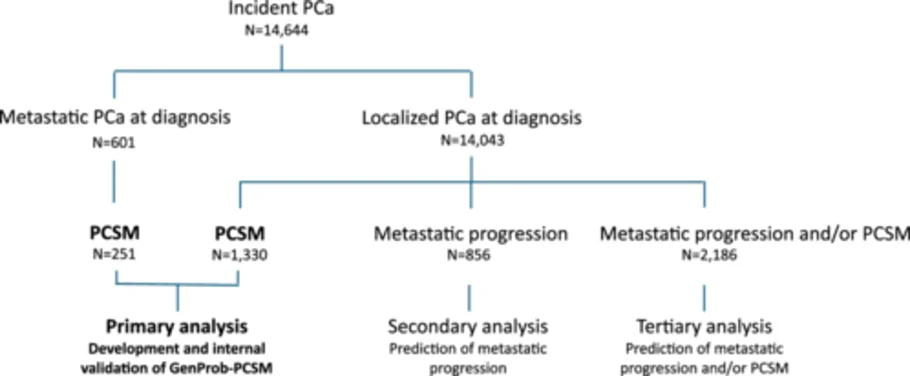
Study design and clinical outcomes in the UK Biobank. A total of 14,644 men with incident prostate cancer (PCa) after UK Biobank recruitment were included. The primary analysis developed and internally validated GenProb‐PCSM using prostate cancer‐specific mortality (PCSM) as the endpoint. The secondary analysis evaluated GenProb‐PCSM for predicting metastatic progression among men with localized PCa at diagnosis after excluding patients with PCSM. The tertiary analysis evaluated the composite endpoint of metastatic progression and/or PCSM. Among the 601 patients with metastatic disease at diagnosis, metastatic disease was recorded before the cancer registry date of PCa diagnosis in 208 patients, likely reflecting administrative differences between hospital records and the cancer registry; these patients were classified as having metastatic disease at diagnosis. [Color figure can be viewed at wileyonlinelibrary.com]

**Table 1:** Characteristics of incident prostate cancer patients included in the UK Biobank (UKB).

Characteristic	Incident prostate cancer in UKB
Incident prostate cancer (PCa), *N* (%)^a^	14,644 (100)
Age diagnosis, years, median (IQR)	69.61 (65.38–73.86)
Genetic ancestry, *N* (%)	
European (NFE, FIN, ASJ)^b^	13,946 (95.23)
Non‐European (remaining)	698 (4.77)
Primary analysis, training	8,786 (60)
Age diagnosis, years, median (IQR)	69.63 (65.35–73.88)
Genetic ancestry, *N* (%)	
European (NFE, FIN, ASJ)^b^	8366 (95.22)
Non‐European (remaining)	420 (4.78)
PCa‐specific mortality (PCSM), *N* (%)	935 (10.64)
Age at PCSM, years, median (IQR)	75.24 (71.23–79.37)
Primary analysis, testing	5,858 (40)
Age diagnosis, years, median (IQR)	69.56 (65.40–73.81)
Genetic ancestry, *N* (%)	
European (NFE, FIN, ASJ)^b^	5580 (95.25)
Non‐European (remaining)	278 (4.75)
PCa‐specific mortality (PCSM), *N* (%)	646 (11.03)
Age at PCSM, years, median (IQR)	75.42 (71.58–79.39)
Secondary analysis	
Localized PCa at diagnosis	14,043 (95.90)
Age diagnosis, years, median (IQR)	69.48 (65.28–73.69)
Metastatic progression	856 (6.10)
Lethal PCa	2186 (15.57)
Metastatic PCa at diagnosis	601 (4.10)
Age diagnosis, years, median (IQR)	73.36 (68.53–77.30)

Abbreviations: IQR, interquartile range; PCSM, prostate cancer‐specific mortality.

a Subjects with missing genotype data and with age dx before recruitment were excluded.

b Subjects with combined probability of > 0.75 for FIN (Finnish), NFE (non‐Finnish European), and ASJ (Ashkenazi Jewish).

### 3.2 Development and Internal Validation of GenProb‐PCSM

The incident PCa cohort was randomly divided into training (60%) and independent testing (40%) cohorts. GenProb‐PCSM was developed in the training cohort as a weighted continuous germline score integrating P/LP variants in the ten NCCN‐recommended DDR genes together with the common *KLK3* I179T variant. Gene‐specific coefficients used to derive the score are presented in Supporting Information Table S1. GenProb‐PCSM translated germline genetic information into a continuous distribution of estimated cumulative PCSM probability by age 75 years (Figure 2). In the training cohort of 8,786 incident PCa patients, the majority of patients clustered below 6%, corresponding primarily to non‐carriers. A distinct elevation immediately above 6% was observed primarily among carriers of the *KLK3* I179T variant (*N* = 981 [11.17%]), including 951 (10.82%) heterozygous and 30 (0.34%) homozygous carriers, whereas progressively higher predicted risks were predominantly driven by carriers of P/LP variants in NCCN‐recommended DDR genes [*N* = 360 (4.10%)], with the highest probabilities observed among carriers of multiple variants (*N* = 30 [0.34%]; Figure 2a). Based on this empirical distribution, predefined cutoffs were established to classify patients into low‐, intermediate‐, and high‐risk groups.

**Figure 2:**
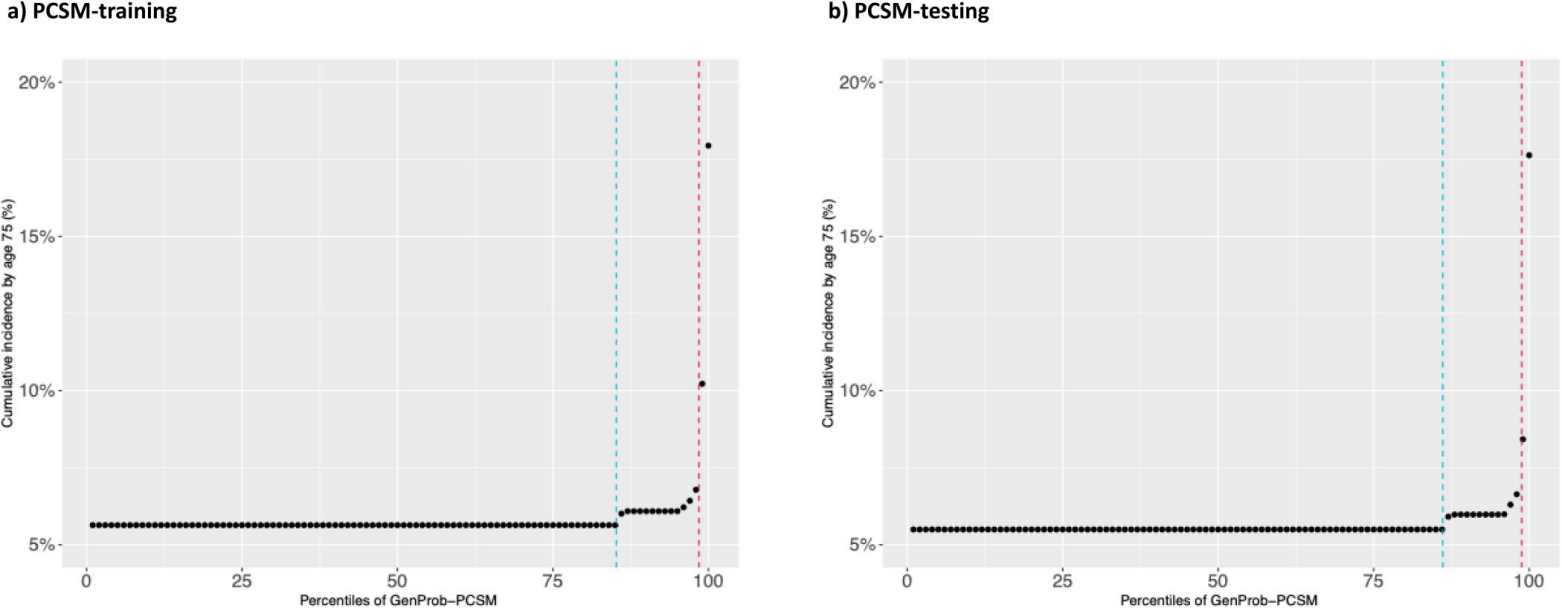
Development and interpretation of GenProb‐PCSM in the training (a) and independent testing (b) cohorts. The estimated cumulative probability of prostate cancer‐specific mortality (PCSM) by age 75 years is shown across percentiles of GenProb‐PCSM, illustrating the relationship between the weighted germline score and inherited risk. For clinical application, estimated PCSM risk was categorized into low‐, intermediate‐, and high‐risk groups using two empirically defined distribution‐based cutoffs after log10 transformation of GenProb‐PCSM. The blue dashed line indicates the cutoff separating the low‐ and intermediate‐risk groups, and the red dashed line indicates the cutoff separating the intermediate‐ and high‐risk groups. The cutoffs were derived in the training cohort and applied without modification to the independent testing cohort. The first cutoff was selected at the density valley separating the two major modes of the distribution, and the second at the midpoint of the largest gap in the upper tail. These empirically defined thresholds provide clinically interpretable risk categories while preserving the continuous representation of inherited risk provided by GenProb‐PCSM. [Color figure can be viewed at wileyonlinelibrary.com]

The predefined model coefficients and risk thresholds derived in the training cohort were subsequently applied without modification to the independent testing cohort. The distribution of estimated cumulative PCSM probability closely resembled that observed in the training cohort (Figure 2b). In both the training and testing cohorts, cumulative incidence curves demonstrated progressive separation of the predefined low‐, intermediate‐, and high‐risk groups (Figure 3a,b). In the independent testing cohort, GenProb‐PCSM remained significantly associated with PCSM both as a continuous predictor (HR per SD, 1.18; 95% CI, 1.12–1.24; *p* < 0.001) and as predefined risk groups. Compared with the low‐risk group, patients in the intermediate‐ and high‐risk groups had HRs of 1.49 (95% CI, 1.22–1.84) and 4.04 (95% CI, 2.58–6.33), respectively (Table 2). Model discrimination was comparable between the training and testing cohorts (C‐index 0.67 vs 0.68), and calibration demonstrated close agreement between predicted and observed cumulative incidence of PCSM by age 75 years in both cohorts (Supporting Information Figure 1a,b).

**Figure 3:**
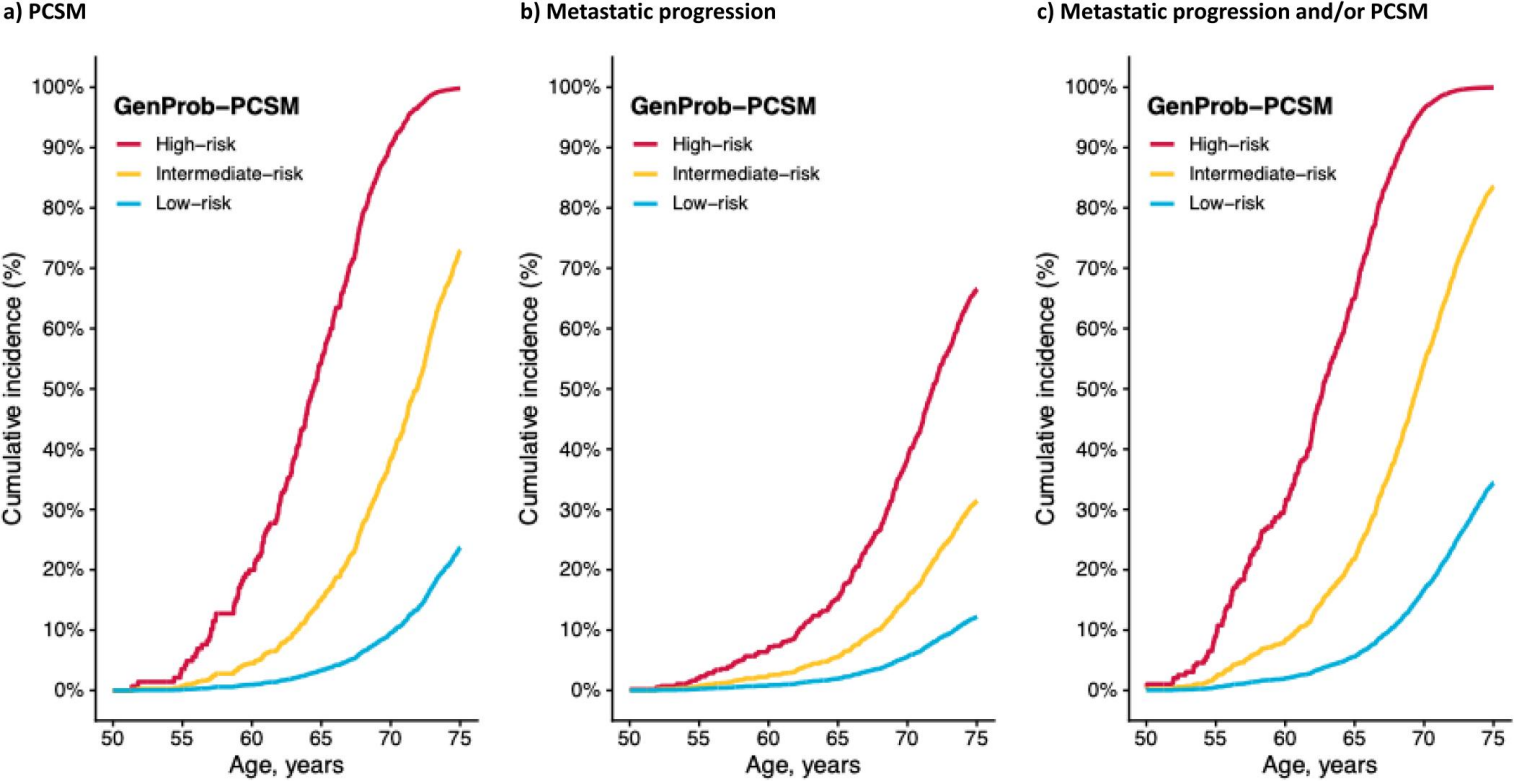
Risk stratification by GenProb‐PCSM. Cumulative incidence curves according to GenProb‐PCSM risk groups for (a) prostate cancer‐specific mortality (PCSM), (b) independent metastatic progression among men with localized PCa at diagnosis after excluding patients with PCSM, and (c) the composite endpoint of metastatic progression and/or PCSM. GenProb‐PCSM stratified patients into low‐, intermediate‐, and high‐risk groups with progressively increasing cumulative probability across all three clinical outcomes. [Color figure can be viewed at wileyonlinelibrary.com]

**Table 2:** Association of GenProb‐PCSM and out comes in primary, secondary, and tertiary analysis.

	Primary analysis			
	Subjects, No. (%)	PCSM, *N*. (per 100,000 person‐year)	HR (95% CI)^a^	*p* value^a^
Training	8786 (60)	935 (1317)		
GenProb‐PCSM, per SD			1.17 (1.13–1.23)	1.10E–13
Low‐risk	7483/8786 (85.17)	755 (1237)	1.00 (ref)	
Intermediate‐risk	1169/8786 (13.31)	143 (1579)	1.20 (1.01–1.44)	0.04
High‐risk	134/8786 (1.53)	37 (4033)	3.42 (2.43–4.83)	2.50E–12
Testing	5858 (40)	646 (1356)		
GenProb‐PCSM, per SD	.	.	1.18 (1.12–1.24)	9.90E–11
Low‐risk	5045/5858 (86.12)	515 (1244)	1.00 (ref)	
Intermediate‐risk	743/5858 (12.68)	108 (1870)	1.49 (1.22–1.84)	1.30E–04
High‐risk	70/5858 (1.19)	23 (4995)	4.04 (2.58–6.33)	1.20E–09

	**Secondary analysis**			
	Subjects, No. (%)	Mets, *N*. (per 100,000 person‐year)	HR (95% CI)	*p* value
Total	12,713 (100)	856 (798)		
GenProb‐PCSM, per SD	.	.	1.09 (1.04–1.15)	3.30E–04
Low‐risk	10,980/12,713 (86.37)	703 (754)	1.00 (ref)	
Intermediate‐risk	1596/12,713 (12.55)	136 (1051)	1.35 (1.12–1.62)	1.40E–03
High‐risk	137/12,713 (1.08)	17 (1576)	2.16 (1.32–3.53)	2.10E–03

	**Tertiary analysis**			
	Subjects, No. (%)	Composite endpoint, *N*. (per 100,000 person‐year)	HR (95% CI)	*p* value
Total	14,043 (100)	2186 (1,963)		
GenProb‐PCSM, per SD	.	.	1.15 (1.11–1.18)	< 1E–300
Low‐risk	12,043/14,043 (85.76)	1766 (1829)	1.00 (ref)	
Intermediate‐risk	1810/14,043 (12.89)	350 (2577)	1.35 (1.21–1.52)	2.50E–07
High‐risk	190/14,043 (1.35)	70 (5831)	3.11 (2.42–3.99)	< 1E–300

^a^ Fine‐Gray models accounting for competing risks (death) using age as the time scale, with left‐truncation at age of diagnosis, and adjusted for top 10 PCs.

### 3.3 GenProb‐PCSM Predicts Independent Metastatic Progression

As a prespecified secondary analysis, we evaluated whether GenProb‐PCSM predicts independent progression to metastatic disease among men initially diagnosed with localized tumors. Therefore, analyzes were restricted to patients with localized PCa at diagnosis and used a mutually exclusive metastatic progression endpoint independent of PCSM. After a median follow‐up of 7.99 years, 856 men developed metastatic progression in the absence of PCSM. Increasing GenProb‐PCSM was significantly associated with an elevated risk of metastatic progression (HR per SD increase, 1.09; 95% CI, 1.04–1.15; *p* < 0.001) (Table 2). When categorized into low‐, intermediate‐, and high‐risk groups derived from the PCSM training model, HRs for metastatic progression were 1.35 (95% CI, 1.12–1.62) and 2.16 (95% CI, 1.32–3.53) for the intermediate‐ and high‐risk groups, respectively, compared with the low‐risk group. In contrast, when the previously published tier‐based framework was applied to metastatic progression, Tier‐1, Tier‐2, and *KLK3* each demonstrated increased risks, although not all reached statistical significance (Supplementary Table 2).

### 3.4 GenProb‐PCSM Predicts the Composite Endpoint

As a prespecified tertiary analysis, we evaluated whether GenProb‐PCSM predicts the composite endpoint of metastatic progression and/or PCSM. After 7.56 years of median follow‐up, 2,186 men developed the composite endpoint. Similarly, increasing GenProb‐PCSM was significantly associated with an elevated risk of the composite endpoint (HR per SD increase, 1.15; 95% CI, 1.11–1.18; *p* < 0.001) (Table 2). When categorized into low‐, intermediate‐, and high‐risk groups derived from the PCSM training model, HRs for the composite endpoint were 1.35 (95% CI, 1.21–1.52) and 3.11 (95% CI, 2.42–3.99) for the intermediate‐ and high‐risk groups, respectively, compared with the low‐risk group.

## 4 Discussion

In this study, we developed and internally validated GenProb‐PCSM, a weighted germline score integrating P/LP variants in the 10 NCCN‐recommended DDR genes together with the common *KLK3* I179T variant into a single inherited risk score for PCSM. GenProb‐PCSM demonstrated robust discrimination and calibration for predicting PCSM and further predicted independent metastatic progression and the composite endpoint of metastatic progression and/or PCSM. To our knowledge, this is the first germline prognostic score developed using PCSM that also significantly predicts metastatic progression among men initially diagnosed with localized PCa, supporting the concept that inherited germline susceptibility contributes across the continuum of lethal disease. Importantly, whereas current germline testing in PCa is primarily used to identify hereditary cancer syndromes and guide treatment selection [22, 23, 24], GenProb‐PCSM expands the clinical utility of germline testing by providing prognostic assessment of inherited susceptibility to lethal disease. Early identification of patients at increased inherited risk of metastatic progression and PCSM may help guide treatment selection, surveillance intensity, and enrollment in clinical trials.

A major strength of this study is the internal validation strategy. Rather than evaluating GenProb‐PCSM solely in the cohort used for model development, we randomly divided the incident PCa cohort into independent training and testing datasets. Gene‐specific weights and clinically interpretable risk thresholds were derived exclusively in the training cohort and successfully validated in the testing cohort, demonstrating the robustness and reproducibility of the score and addressing concerns regarding potential optimism associated with model development.

Compared with our previously published tier‐based germline classification [14], GenProb‐PCSM offers three major advantages. First, GenProb‐PCSM is easier to use in clinical practice because it transforms three separate genetic components (Tier‐1, Tier‐2, and *KLK3*) into a single inherited risk score. This simplified method reduces the complexity of interpreting multiple germline findings and facilitates integration into routine clinical practice. A single inherited risk score is easier for clinicians to interpret, communicate, and incorporate into clinical decision‐making than multiple categorical genetic components. As germline testing becomes increasingly incorporated into PCa care, simplifying the interpretation of inherited genetic information may lower an important barrier to broader clinical adoption.

Second, GenProb‐PCSM provides a more accurate representation of inherited risk by preserving gene‐specific effects. Unlike the tier‐based framework, which assigns equal weights to all genes within the same tier [14], GenProb‐PCSM assigns weights according to the estimated association of each gene with PCSM. This approach preserves biological heterogeneity across NCCN‐recommended DDR genes, whose contributions to PCSM risk differ substantially (Supporting Information Table S1). Because GenProb‐PCSM and the tier‐based framework are derived from essentially the same underlying germline genetic information, only modest improvements in predictive performance are expected. Consistent with this, GenProb‐PCSM demonstrated a modest improvement in discrimination compared with the tier‐based framework (C‐Index, 0.70 vs 0.69). Nevertheless, gene‐specific weighting provides a more biologically faithful representation of inherited susceptibility while preserving the simplicity of a single inherited risk score.

Third, GenProb‐PCSM provides a statistically efficient framework for evaluating inherited susceptibility across related clinical outcomes. By representing germline risk as a single continuous predictor rather than separate Tier‐1, Tier‐2, and *KLK3* variables, GenProb‐PCSM reduces model complexity and degrees of freedom while aggregating the cumulative effects of multiple rare and common variants. This parsimonious representation may improve statistical power for detecting associations with less frequent but clinically important outcomes. Consistent with this concept, all three components of the previous tier‐based framework demonstrated increased risks of metastatic progression, although not all reached statistical significance (Supporting Information Table S2). In contrast, GenProb‐PCSM was significantly associated with metastatic progression, illustrating the advantage of aggregating multiple individually rare genetic effects into a single weighted continuous predictor.

Beyond the internal validation, GenProb‐PCSM also demonstrated predictive utility across related clinical outcomes. Although the score was developed using PCSM in the training cohort and internally validated in the independent testing cohort, it was subsequently evaluated in a distinct analytic population restricted to men with localized PCa at diagnosis using metastatic progression as a mutually exclusive clinical endpoint independent of PCSM. Because no patients with PCSM were included in this secondary analysis, the significant association observed provides additional evidence that GenProb‐PCSM predicts an earlier manifestation of inherited susceptibility to lethal disease. Furthermore, its significant association with the composite endpoint extends the clinical utility of GenProb‐PCSM beyond PCSM and supports the concept that germline genetic predisposition contributes across the continuum of lethal disease, from metastatic progression to PCSM [5, 6].

GenProb‐PCSM may also complement tissue‐based prognostic classifiers, such as Decipher, rather than replace them [22, 25]. Tissue‐based assays characterize the molecular features of an established tumor and are typically obtained after biopsy or surgery, whereas GenProb‐PCSM measures inherited susceptibility that is fixed from birth and can be available before diagnosis, at diagnosis, and throughout the disease course. Thus, GenProb‐PCSM may provide prognostic information that is biologically distinct from tumor‐derived classifiers, particularly by identifying patients with an inherited predisposition to metastatic progression and PCSM. In clinical practice, GenProb‐PCSM could be used at or before diagnosis to identify patients with an inherited predisposition to aggressive disease, whereas tissue‐based classifiers such as Decipher may provide complementary prognostic information after biopsy or surgery to further refine treatment decisions. Future studies should evaluate whether combining germline and tissue‐based prognostic information improves risk stratification and clinical decision‐making beyond either approach alone.

The present study also aligns with recent recommendations from ACMG advocating a transition from binary carrier status toward quantitative assessment of inherited disease risk [16]. Rather than classifying individuals according to the presence or absence of PVs, GenProb‐PCSM integrates the cumulative effects of multiple rare and common germline variants into a single continuous measure of inherited susceptibility. In this regard, GenProb‐PCSM provides a practical implementation of the ACMG framework for quantitative genomic risk assessment and demonstrates how complex germline findings can be translated into a clinically interpretable inherited risk score. This conceptual approach may serve as a model for future germline prognostic tools in PCa and other diseases.

Several limitations should be acknowledged. First, although GenProb‐PCSM was internally validated using independent training and testing cohorts within the UKB, external validation in independent cohorts with diverse clinical characteristics is needed to confirm its generalizability. Second, the UKB predominantly includes individuals of European ancestry, and additional studies are needed to evaluate performance across other ancestral populations. Third, although GenProb‐PCSM predicts clinically important outcomes beyond PCSM, its incremental value when combined with established clinicopathologic prognostic factors, including tumor grade, stage, PSA level, and genomic tumor classifiers, warrants further investigation. Fourth, prospective studies are needed to determine whether incorporating GenProb‐PCSM into clinical decision‐making improves patient management and outcomes. Finally, GenProb‐PCSM was developed using established germline predictors of PCSM, including NCCN‐recommended DNA damage repair genes and the *KLK3* I179T variant. Future studies should evaluate whether incorporating additional validated germline markers from other biological pathways, such as androgen signaling, apoptosis, and immune regulation, further improves prognostic performance.

In conclusion, GenProb‐PCSM transforms complex germline findings into a single inherited risk score that captures inherited susceptibility across the continuum of lethal prostate cancer. By combining simplicity, gene‐specific weighting, and robust predictive performance, GenProb‐PCSM provides a practical framework for implementing germline prognostic assessment in PCa.

## Funding

The authors have nothing to report.

## Conflicts of Interest

Endeavor Health has agreements with GenomicMD and GoPath Diagnostics for genetic tests of polygenic risk scores. J. Xu serves a scientific advisory board member for GoPath Diagnostics and GenomicMD.

## Supporting information


**Figure S1:** Calibration of GenProb‐PCSM in the training and testing cohorts. Calibration plot comparing predicted and observed cumulative incidence of prostate cancer‐specific mortality (PCSM) by age 75 years across deciles of predicted risk in the (a) training cohort and (b) independent testing cohort. Each circle represents one decile of predicted risk. GenProb‐PCSM demonstrated good calibration in both cohorts, with close agreement between predicted and observed cumulative incidence of PCSM.

Supporting File

## Data Availability

The data that support the findings of this study are available from the corresponding author upon reasonable request.
